# Superbug: The Fatal Menace of MRSA

**DOI:** 10.3201/eid1610.101108

**Published:** 2010-10

**Authors:** James P. Steinberg

**Affiliations:** Author affiliation: Emory University Hospital Midtown, Atlanta, Georgia, USA

**Keywords:** Bacteria, Staphylococcus aureus, MRSA, infections, antimicrobial resistance, methicillin resistance, book review

This book is an extensively researched and detailed review of methicillin-resistant *Staphylococcus aureus* (MRSA) by Maryn McKenna, a journalist and the former Centers for Disease Control and Prevention beat reporter for the Atlanta Journal Constitution. Although McKenna has a background in science reporting, she infused this work with drama, an approach that will draw in some readers but be off-putting to others. To the reader familiar with MRSA, the use of hyperbole coupled with factual inaccuracies leaves one wondering where truth stops and fiction begins. These shortcomings may keep this work off scholarly reading lists.

Most chapters include case presentations that emphasize the emotional toll wrought by MRSA infections. The cases effectively introduce topics such as MRSA in athletes and other risk groups, MRSA in animals, and postinfluenza MRSA pneumonia. The chapter on infections caused by the 80/81 strain of *S. aureus *in the 1950s is particularly useful because it demonstrates parallels between the 1950s epidemic and the USA300 clone of MRSA today. However, McKenna infers that the 80/81 strain disappearance was caused by use of antistaphylococcal drugs and not natural events. Although 80/81 did disappear after the introduction of methicillin, the cause of the strain’s disappearance is largely unknown. 

The community-onset MRSA epidemic of the past decade is not presented with a clear timeline. As a result, the reader is unclear if the incidence of disease is still increasing, has leveled, or is decreasing and could further parallel incidence of the 80/81 strain.

The chapter on healthcare-associated infections is MRSA centric and misses excellent opportunities to frame these infections and problems such as antimicrobial drug resistance and overuse in a broader context. The challenges of MRSA prevention are not balanced with other infection prevention priorities such as control of multidrug-resistant gram-negative pathogens and *Clostridium difficile*. Active surveillance to identify MRSA carriers is emphasized more than hand hygiene. Legislation mandating MRSA screening is discussed without explaining why major infection prevention organizations believe such legislation is unwise.

Some of the physicians, researchers, and other heroes in the MRSA story are appropriately praised. Failings of physicians and the healthcare establishments are deservedly criticized. However, there is no call to arms over some of the most egregious medical failures, such as poor hand hygiene compliance and unwise antimicrobial drug use. The reader is left frustrated about the inability of the medical establishment to control MRSA.

The book attempts to appeal to a broad audience, and although McKenna uses a lot of medical jargon, she effectively explains concepts such as antimicrobial drug mechanisms and molecular typing. Her style and the human interest stories will appeal to a lay audience, particularly consumer advocates. The historical background and scientific detail may appeal to healthcare professionals interested in infectious diseases or public health. However, the main goal of the book appears to be to scare the reader about the “Superbug.” In this regard, McKenna succeeds. 

